# Efficacy and safety of vonoprazan-amoxicillin dual therapy versus bismuth-containing quadruple therapy for patients with *Helicobacter pylori* infection: a meta-analysis

**DOI:** 10.3389/fmicb.2025.1561749

**Published:** 2025-03-19

**Authors:** Xiao Li, Cheng Jiang, Yuwen Su, Ruiyun Gao, Peijun Yang, Yuechen Qin, Yue Zou, Weiming Liang, Jieru Quan, Liying Pan

**Affiliations:** ^1^The First Affiliated Hospital of Guangxi University of Science and Technology, Guangxi University of Science and Technology, Liuzhou, Guangxi, China; ^2^Lingui Campus, Guilin Medical University, Guilin, Guangxi, China; ^3^School of Economics and Management, Guangxi University of Science and Technology, Liuzhou, Guangxi, China

**Keywords:** *H. pylori*, amoxicillin, vonoprazan, eradication, bismuth-containing quadruple therapy, compliance, adverse event, meta-analysis

## Abstract

**Introduction:**

This meta-analysis aims to compare the efficacy and safety of vonoprazan-amoxicillin (VA) dual therapy in comparison to bismuth-containing quadruple therapy (BQT) for patients with *Helicobacter pylori* (*H. pylori*) infection.

**Materials and methods:**

Four databases (PubMed, Embase, Web of Science, and Cochrane Library) were searched published from establishment of database to June 1, 2024, for articles studying VA dual therapy compared to BQT for patients with *H. pylori* infection. Meta-analyses of eradication rates, adverse events, compliance and cost were preformed.

**Results:**

A total of 17 studies were included for meta-analysis. Compared with BQT, VA increased the incidence of *H. pylori* eradication rate, with significant difference under the ITT analysis (86.9% vs. 80.4%, RR = 1.07, 95% CI: 1.01–1.12, *p* = 0.01) but there no significant difference under the PP analysis (90.7% vs. 86.5%, RR = 1.03, 95% CI: 0.99–1.08, *p* = 0.13). Besides, VA significantly increased compliance (RR = 1.03, 95% CI: 1.01–1.05, *p* < 0.01) and decreased the occurrence of total adverse events (27.0% vs. 11.5%, RR = 0.43, 95% CI: 0.37–0.51, *p* < 0.01). Furthermore, VA has lower cost compared to BQT.

**Conclusion:**

Our findings indicated that VA dual therapy provided a higher eradication rate, enhanced compliance, decreased adverse events, and lowered cost relative to BQT for patients with *H. pylori* infection.

**Systematic review registration:**

https://www.crd.york.ac.uk/prospero/display_record.php?ID=CRD42024576738, identifier CRD42024576738 (PROSPERO).

## 1 Introduction

*Helicobacter pylori* is a Gram-negative bacterium that infiltrates the mucosa of the stomach, infecting almost 50% of the global population (Shatila and Thomas, 2022; Sharma et al., 2024). *H. pylori* is often first acquired throughout childhood and remains present indefinitely unless it is treated (Ibrahim, 2024). *H. pylori* causes stomach mucosal injury and is connected to duodenal and gastric ulcers, gastric adenocarcinoma, and MALT lymphoma (Sugano et al., 2015; Malfertheiner et al., 2017). Ten to 15 % of peptic ulcers and less than 1% of gastric adenocarcinoma have *H. pylori* infection (Kanu and Soldera, 2024). Besides, it will also cause 90% of non-cardia stomach cancer and 5% of all malignancies (Moss, 2017). The elimination of *H. pylori* effectively treats gastritis and has the potential to modify the course toward long-term problems or the reoccurrence of the disease (Malfertheiner et al., 2017). A thorough removal of *H. pylori* has reduced stomach cancer and other disorders including Epstein–Barr virus (Grantham et al., 2023). Moreover, *H. pylori* can causes some extragastric diseases, such as iron-deficient anemia, vitamin B12 deficiency, chronic immune thrombocytopenia (cITP), metabolic syndrome, non-alcoholic fatty liver disease, Alzheimer’s, neurological illnesses, and cardiovascular diseases (Takeuchi and Okamoto, 2022; Gravina et al., 2020).

For 30 years, *H. pylori* has been treated using triple therapy—PPI, amoxicillin, and clarithromycin (PPI-AC), but clarithromycin resistance has quickly reduced the clearance rate of PPI-AC to below 80% in some places (Li et al., 2024). In China, where metronidazole, levofloxacin, and clarithromycin resistance rates exceed 15%, BQT is the principal empirical treatment for *H. pylori* eradication (Li et al., 2024). To eliminate *H. pylori*, the sixth Chinese National Consensus Report advises quadruple therapy: bismuth, PPIs, and two antibiotics (He et al., 2024). However, Proton pump inhibitors (PPIs) can reduce gastric acid depending on dosage and the host’s CYP2C19 gene polymorphism, which may affect *H. pylori* eradication (He et al., 2024). BQT has a difficult dosing regimen, frequent side effects, and expensive costs (Li et al., 2024). This medication requires four daily dosage and modest but common adverse events that can affect patient adherence (Katelaris et al., 2023). Thus, new convenient, cost-effective, and treatments with fewer antibiotics and side effects are needed.

The use of potassium-competitive acid blockers (PCABs), such vonoprazan, as substitutes for PPIs in the treatment of *H. pylori* eradication is evolving. In contrast to PPIs, PCABs are not affected by CYP2C19 polymorphisms and offer a more reliable and strong suppression of stomach acid (Katelaris et al., 2023). Vonoprazan (VPZ), which received approval in Japan in 2015 for the treatment of *H. pylori*, functions by blocking H+/K+-ATPase in gastric parietal cells (Okubo et al., 2020). Its extended duration of action, high pKa (9.37), and strong affinity (Ki = 3.0 HM) allow it to provide 24-h acid control during infection (Scott et al., 2015). In a dose-dependent way, successfully inhibits the typical decrease in intragastric pH that occurs during the night (Jenkins et al., 2015). Evidence indicates that VA dual therapy is more effective than PPI-based therapies, and has good safety profiles (Liu et al., 2024). The VA dual therapy regimen has routinely attained eradication rates ranging from 95.6 to 98.5%, which is far higher than the 90% threshold for successful results (Liu et al., 2024). Therefore, medicines based on vonoprazan show significant potential in enhancing the rates of *H. pylori* elimination.

The results of studies comparing the eradication of *H. pylori* with BQT and VA dual therapy are controversial. Thus, we performed a meta-analysis to compare the efficacy and safety of VA versus BQT for patients with *H. pylori* infection.

## 2 Materials and methods

### 2.1 Search strategy

The present meta-analysis was performed using the specific parameters set forth by the Preferred Reporting Project for Systematic Review and Meta-Analysis (PRISMA) 2020 (Tugwell and Tovey, 2021; Swartz, 2021). The present study was officially registered at PROSPERO with the assigned registration number CRD42024576738. A comprehensive search was conducted in the PubMed, Embase, Web of Science, and Cochrane Library databases to identify articles published until June 2, 2024. In the search, the Medical Subject Headings (MeSH) terms “*H. pylori*,” “Vonoprazan,” “bismuth tripotassium dicitrate,” and other pertinent keywords were employed. The specific information regarding the search entries in four databases can be found in Supplementary Table S1. In addition, we conducted a thorough manual examination of the bibliographies of the identified articles, as well as pertinent reviews and meta-analyses, in order to uncover any new studies that fit the criteria for inclusion. Unpublished studies will not be sought.

### 2.2 Inclusion and exclusion criteria

The inclusion criteria are as follows: (1) patients with *H. pylori* infection who have not achieved eradication; (2) patients in the intervention group received VA dual therapy; (3) patients in the control group received BQT; (4) at least one of the following outcomes were reported: *H. pylori* eradication rate, adverse events, compliance and cost of therapy; (5) study design: randomized controlled trial.

The exclusion criteria are as follows: (1) other types of articles, such as editorials, proceeding paper, reviews, trial registry records, abstracts, meta-analyses, retrospective studies; (2) Not relevant; (3) Not consistent with intervention; (4) Not HP positive patients; (5) Duplicate records.

### 2.3 Selection of studies

The literature selection procedure, which included the elimination of duplicate entries, was carried out using EndNote (Version 20; Clarivate Analytics). Two independent reviewers carried out the first search. After removing the redundant content, the relevancy of the titles and abstracts was assessed. Each study was then categorized as either included or removed. We reached a consensus and resolved the issue that way. Should the concerned parties fail to reach a mutually acceptable resolution, a third reviewer steps in as a mediator.

### 2.4 Data extraction

Two independent reviewers extracted data. The extracted data included: (1) Basic characteristics of studies included: (1) author, nationality, year of publication, study design; (2) Baseline characteristics of study subjects: sample size, age, gender, therapeutic schedule, smoking, alcohol, *H. pylori* detect methods; (3) Outcome indicators: eradication rates, nausea and/or vomiting, diarrhea, skin rash, dizziness and/or headaches, abdominal distension, abdominal pain, taste problem, constipation and compliance, cost of therapy. The primary methodologies utilized for the verification of *H. pylori* eradication are as follows: (1) Urea breath test (UBT), a non-invasive and extremely accurate technique, can be used to assess the eradication rate of *H. pylori* at least 4 weeks following the conclusion of treatment. As per the manufacturer’s specifications, a delta over baseline score of 3.5 or above indicates the existence of urease activity linked to *H. pylori* and is considered a positive result (Charach et al., 2024). A negative test result indicated successful eradication, while a positive test result indicated failed eradication. (2) Histopathology. Histological analysis of stomach samples is still a dependable way to find *H. pylori*. If *H. pylori* is found in stained sections, an infection is thought to be diagnosed (Redéen et al., 2011). (3) Rapid urease test (RUT), a quick and simple method to find out the rate at which *H. pylori* is eradicated. A positive RUT necessitates that there be roughly 10^5^ *H. pylori* in the biopsy sample for the color to change (typically to red or pink) due to the urease enzyme of *H. pylori* produces ammonia from urea (Takahiro Uotani, 2015). (4) Culture. Culture of *H. pylori* from gastric biopsies is the gold standard for diagnosis. Typical colonial and Gram stain morphologies, as well as whether the organisms tested positive for oxidase, catalase, and urease, were used to determine the type of organism (Grove et al., 1998).

### 2.5 Risk of bias

The risk of bias was assessed using the Cochrane Risk of Bias tool (Julian Higgins et al., 2020) by two independent reviewers. Each trial was reviewed and scored as having a high, low, or unclear risk of bias according to the following domains: random sequence generation, allocation concealment, blinding of participants and personnel, blinding of outcome assessment, incomplete outcome data, selective reporting and others bias. The controversial results were resolved by group discussion if there were discrepancies.

### 2.6 Statistical analysis

The selection duplicate removal of studies included was conducted using EndNote (Version 20; Clarivate Analytics). All analyses were performed using Review manager 5.3 (Cochrane Collaboration, Oxford, United Kingdom). The comparation of VA dual therapy and BQT was assessed using risk ratios (RR) with corresponding 95% confidence intervals (CI), and collected the data of the *H. pylori* eradication rate, overall adverse events, adverse events, compliance, relevant cost of therapy. When selecting the specific treatment plan and sample size of a type of therapy, if there are two groups of the same type of therapy, the sample size and specific treatment plan of the group with better curative effect are selected. Statistical heterogeneity between included studies was calculated using the *Q*-tests and *I*^2^ statistic (*I*^2^ scores of 0–50% signify low heterogeneity, and values higher than 50% demonstrate high heterogeneity). When the inter-study heterogeneity is high, the random effects model is used, otherwise the fixed effects model is used (Julian Higgins et al., 2020). *P-*value <0.05 was considered statistically significant. The cost divided by the eradication rate can produce the cost-effectiveness ratio (CER) for various treatment approaches. The objective of the cost-effectiveness study is to examine therapy alternatives that are more economical in attaining a specified therapeutic outcome. A greater CER signifies that the treatment choice was less cost-effective.

## 3 Results

### 3.1 Literature search

**Figure 1:**
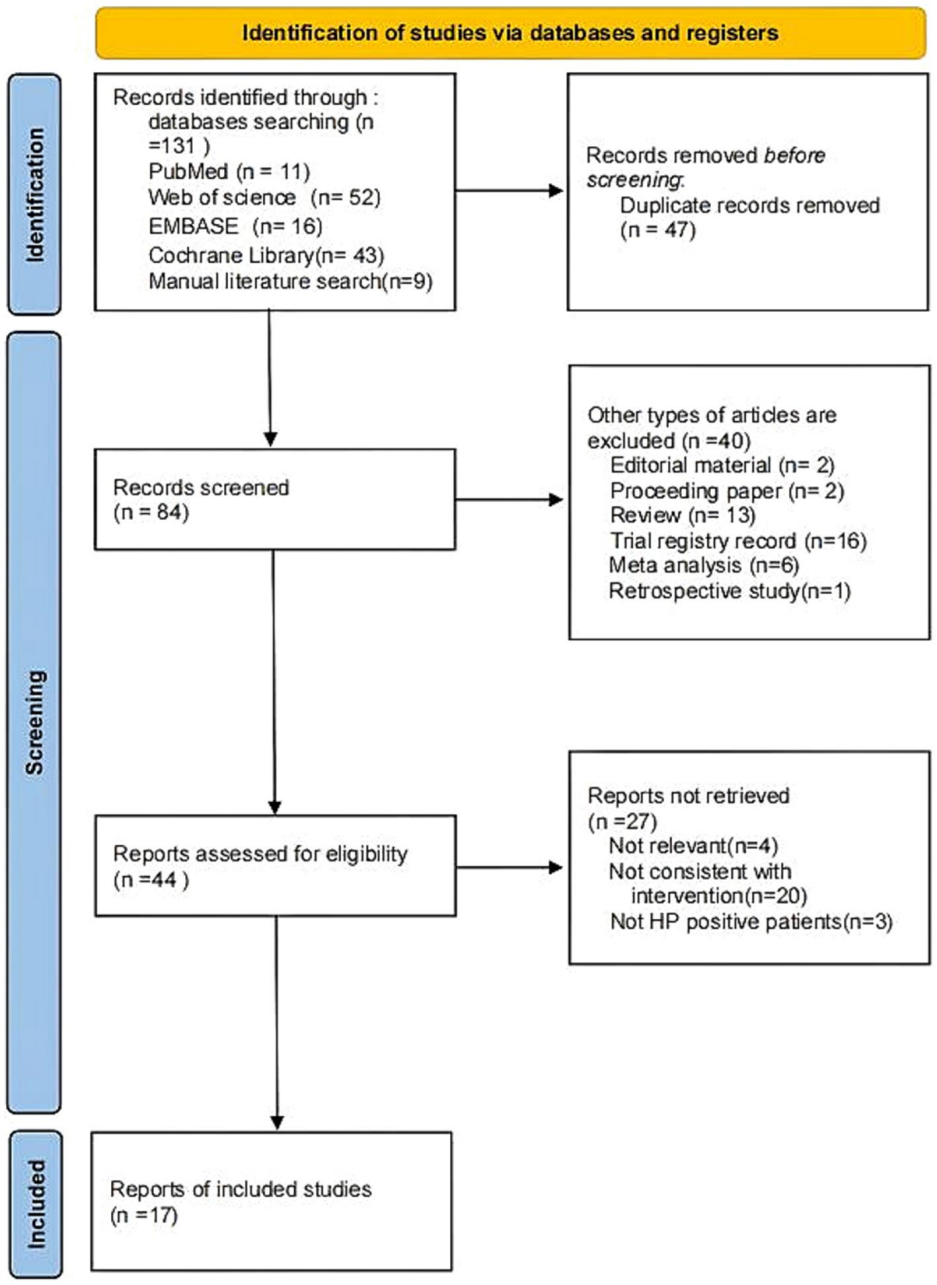
Flow chart of literature search strategies.

A total of 131 records were initially retrieved from the PubMed, Web of science, Embase, Cochrane databases and manual literature search with 11, 52, 16, 43, and 9 records, respectively. Forty-seven duplicate records were removed, and 67 records were excluded based on title and abstract screening. In the second stage, the full-text of 17 studies was reviewed, to make sure they meet the criteria for inclusion. Finally, a total of 17 RCTs (Chen et al., 2024; Chen et al., 2022; Meng et al., 2023; Hu et al., 2023; Huangling et al., 2022; Cheung Ka et al., 2024; Fujin, 2023; Li et al., 2023; Gaozhong et al., 2023; Guohua et al., 2024; Peng et al., 2023; Qian et al., 2023; Ratana-Amornpin et al., 2023; Wang et al., 2023; Ting et al., 2023; Liang et al., 2023; Yang et al., 2023) were included in this analysis. The selection process of the studies was present in Figure 1.

### 3.2 Study characteristics

Table 1 summarized the basic characteristics of the 17 RCTs. All of the RCTs were published after 2022, with three (Chen et al., 2024; Cheung Ka et al., 2024; Guohua et al., 2024) published in 2024. Sixteen RCTs were performed in China and one (Ratana-Amornpin et al., 2023) in Thailand. The sample size spanned from 47 to 400. The detail information of country, age, gender, smoking, alcohol, sample size, regimen and method of *H. pylori* detect was present in Table 1. In addition, four studies (Chen et al., 2024; Li et al., 2023; Liang et al., 2023; Yang et al., 2023) reported the cost spent on treatment regimens.

**Table 1:** Characteristics of included studies.

Study	Country	Design	Age (mea*n*, SD)	Gender Male%	Smoking/alcohol	Group	No	Regimen	*H. pylori* detect
Chen et al. (2024)	China	RCT	44.87 ± 2.00	44.4%	N/N	VA	45	V 20 mg bid, A 1.0 g tid, 14 days	UBT/UBT
			40.78 ± 1.49	55.6%	N/N	BQT	45	l 5 mg, A 1.0 g, F 100 mg, B 240 mg, bid, 14 days	
Chen et al. (2022)	China	RCT	41.52 ± 11.34	47.62%	N/N	VA	63	V 20 mg bid, A 500 mg tid, 7 days	UBT/UBT
			40.67 ± 12.41	46.03%	N/N	BQT	63	E 20 mg bid, A 1.0 g bid, C 0.5 g bid, CPB 150 mg qid, 14 days	
Meng et al. (2023)	China	RCT	42.5 ± 11.5	43.10%	13.79%/20.70%	VA	58	V 20 mg bid, A 750 mg tid, 14 days	UBT, H/UBT
			47.8 ± 15.3	43.86%	17.54%/12.30%	BQT	57	E 40 mg, qd, A 1000 mg, bid, F 100 mg, bid, CB 150 mg, tid, 14 days	
Hu et al. (2023)	China	RCT	/	34.02%	14.43%/44.33%	VA	97	V 20 mg bid, A 1000 mg tid, 14 days	UBT, RUT/UBT
			/	42.27%	13.40%/43.30%	BQT	97	E 20 mg bid, B 220 mg bid, A 1,000mg bid, M 0.4 g qid, 14 days	
Huangling et al. (2022)	China	RCT	47.62 ± 4.38	57.00%	N/N	VA	100	V 20 mg bid, A 1 g bid, 14 days	UBT, H/UBT
			47.70 ± 4.42	59.00%	N/N	BQT	100	O 20 mg bid, B 0.6 mg bid, A 1,000 mg bid, F, 20 mg tid, 14 days	
Cheung Ka et al. (2024)	China	RCT	35.9 ± 8.3	45.00%	15.00%/16.7%	VA	100	V 20 mg bid, A 1 g tid, 14 days	UBT/UBT
			35.0 ± 7.4	44.00%	17.00%/20.7%	BQT	100	B 220 mg bid, E 20 mg bid, Te 500 mg tid, M 500 mg qid, 14 days	
Fujin (2023)	China	RCT	49.44 ± 9.50	46.67%	N/N	VA	60	V 20 mg qd, A 1 g bid, 14 days	UBT/UBT
			48.72 ± 9.31	48.33%	N/N	BQT	60	R 20 mg bid, B 220 mg bid, A 1000 mg bid, C 500 mg bid, 14 days	
Li et al. (2023)	China	RCT	45.85 ± 13.97	34.67%	42.67%/85.33%	VA	75	V 20 mg bid, A 750 mg tid, 14 days	UBT, RUT/UBT
			42.67 ± 12.61	45.33%	49.33%/69.33%	BQT	75	E 20 mg bid, B 220 mg bid, A 1000 mg bid, F 100 mg bid, 14 days	
Gaozhong et al. (2023)	China	RCT	/	46.15%	/	VA	65	V 20 mg bid, A 0.75 g q6h, 14 days	UBT/UBT
			/	50.77%	/	BQT	65	V20mg bid, A1.0 bid, L0.5g qd or C0.5 g bid or M0.4g q6h, Bs220mg bid, 14 days	
Guohua et al. (2024)	China	RCT	44.6 ± 8.7	41.70%	/	VA	100	V 20 mg bid, A 1 g tid, 14 days	UBT/UBT
			43.8 ± 9.3	45.16%	/	BQT	100	E 20 mg bid, B 220 mg bid, A 1 g bid, C 0.5 g bid, 14 days	
Peng et al. (2023)	China	RCT	40.1 ± 12.1	48.10%	12.03%/5.70%	VA	158	V 20 mg bid, A 750 mg qid, 14 days	UBT, H/UBT
			41.9 ± 12.6	44.94%	10.76%/6.96%	BQT	158	E 20 mg bid, CBS 220 mg bid, A 1000 mg bid, C 500 mg bid, 14 days	
Qian et al. (2023)	China	RCT	41.04 ± 14.87	50.40%	16.80%/34.40%	VA	125	V 20 mg bid, A 1000 mg bid, 10 days	UBT, RUT, H/UBT
			43.30 ± 13.93	47.20%	17.60%/31.20%	BQT	125	E 20 mg bid, CBP 200 mg bid, A 1000 mg bid, C 500 mg bid, 10 days	
Ratana-Amornpin et al. (2023)	Thailand	RCT	50.4 ± 13.5	57.14%	4.76%/4.76%	VA	21	V 20 mg bid, A 500 mg qid, 14 days	RUT, H, culture/UBT
			55.5 ± 14.2	42.31%	0.00%/0.00%	BQT	26	O 20 mg bid, Bs 1,048 mg bid, A 1000 mg bid, C-MR 1 g qd, 14 days	
Wang et al. (2023)	China	RCT	44.2 ± 10.8	48.6%	6.8%/33.8%	VA	74	V 20 mg bid, A 750 mg qid, 14 days	UBT/UBT
			44.5 ± 8.8	44.2%	13.0%/29.9%	BQT	77	R 10 mg bid, B 220 mg bid, A 1000 mg bid, C 500 mg bid, 14 days	
Ting et al. (2023)	China	RCT	72.5 ± 5.8	61.67%	/	VA	60	V 20 mg bid, A 500 mg tid, 7 days	UBT/UBT
			73.6 ± 5.9	55.00%	/	BQT	60	O 20 mg bid, B 110 mg tid, A 1000 mg bid, C 500 mg bid, 14 days	
Liang et al. (2023)	China	RCT	28.2 ± 7.2	80.95%	/	VA	42	V 500 mg bid, A 1 g tid, 14 days	UBT, H/UBT
			27.7 ± 6.6	79.07%	/	BQT	43	E 20 mg bid, B 220 mg bid, A 1000 mg bid, F100 mg bid, 14 days	
Yang et al. (2023)	China	RCT	48.92 ± 11.9	52.00%	20.50%/14.50%	VA	200	V 20 mg bid, A 1000 mg tid, 10 days	UBT/UBT
			46.01 ± 11.7	49.00%	15.00%/21.00%	BQT	200	R 20 mg bid, B/T/C, combined package 4.2 g, bid, 14 days.	

V, vonoprazan; A, amoxicillin; F, furazolidone; C, clarithromycin; T, tinidazole; M, metronidazole; Te, tetracycline; L, levofloxacin; O, omeprazole; E, esomeprazole; R, rabeprazole; I, ilaprazole; B, bismuth potassium citrate; CPB, colloidal pectin bismuth; CB, colloidal bismuth; CBS, colloidal bismuth subcitrate; Bs, bismuth subsalicylate; CBP, colloidal bismuth pectin; Ba, bismuth agent; UBT, urea breath test; culture: *H. pylori* culture; RUT, rapid urease test; H, histopathology.

### 3.3 Risk of bias

**Figure 2:**
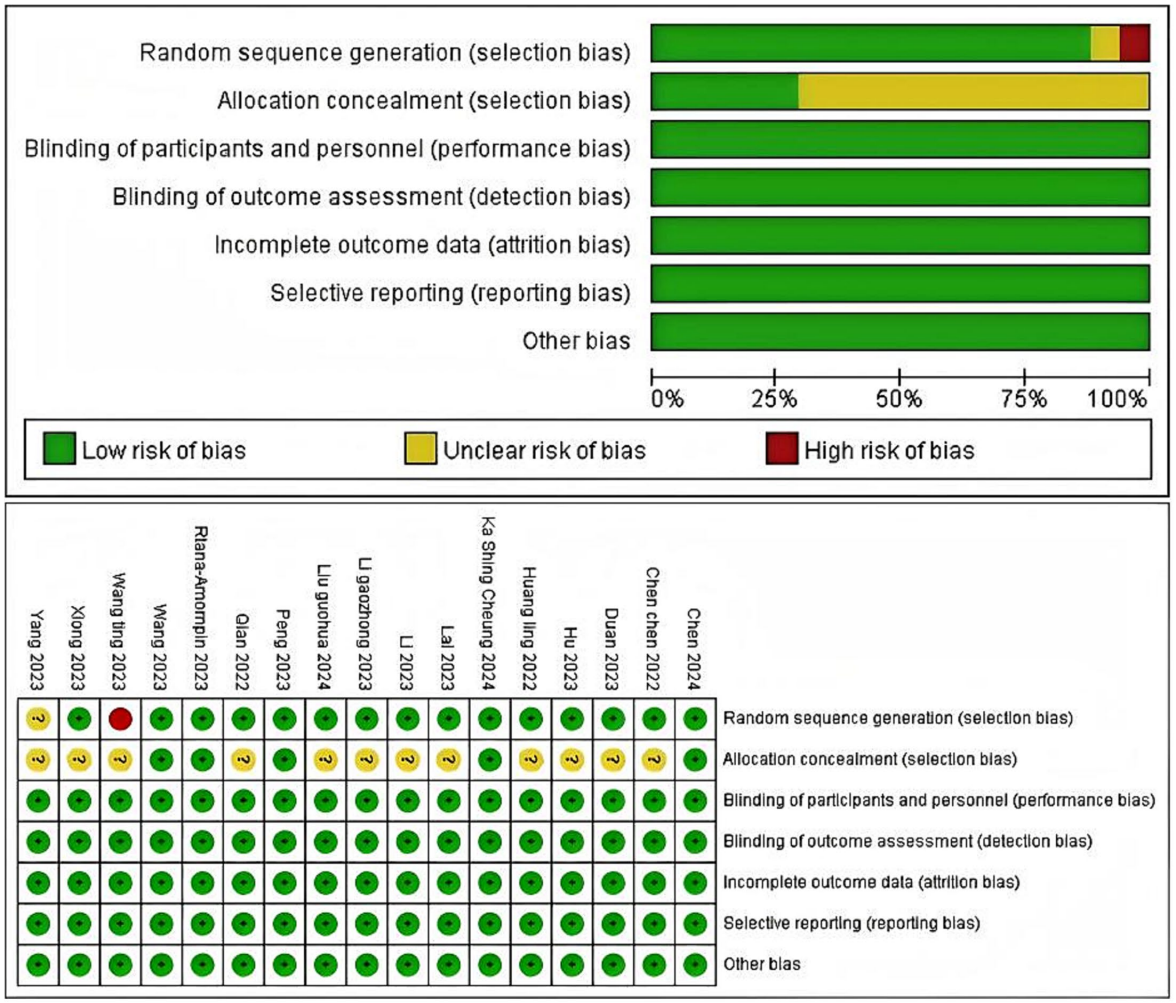
Risk of bias assessment for the included RCTs.

According to the Cochrane risk of bias tool, of the 17 studies, 15 studies produced sufficient random sequences, 5 studies reported adequate allocation concealment, and 12 studies had unclear allocation concealment. Seventeen studies had complete outcome data, reported no selectivity, and had no other bias. As for blinding, the risk was low in all the included studies. The results of the assessment are summarized in Figure 2.

### 3.4 Primary outcomes

Table 2 showed results of meta-analysis for all clinical outcomes.

**Table 2:** Results of meta-analysis for all clinical outcomes.

Outcomes	No. of studies	Sample size		Heterogeneity		Overall effect size	95% CI of overall effect	*P*-value
		VA	BQT	*I*^2^ (%)	*P*-value			
*H. pylori* eradication rate (ITT)	17	1,443	1,451	62	<0.01	RR = 1.07	1.01 ~ 1.12	0.01
*H. pylori* eradication rate (PP)	17	1,369	1,324	5	<0.01	RR = 1.03	0.99 ~ 1.08	0.13
Overall AEs	16	1,310	1,309	0	0.99	RR = 0.43	0.37 ~ 0.51	<0.01
Compliance	17	1,440	1,444	46	0.02	RR = 1.03	1.01 ~ 1.05	<0.01
Nausea and/or vomiting	15	1,236	1,232	4	0.41	RR = 0.38	0.26 ~ 0.55	<0.01
Diarrhea	14	1,207	1,210	4	0.39	RR = 0.75	0.55 ~ 1.03	0.08
Skin rash	10	833	831	6	0.64	RR = 0.76	0.42 ~ 1.38	0.36
Dizziness and/or headaches	7	777	777	0	0.65	RR = 0.33	0.13 ~ 0.84	0.02
Abdominal distension	7	489	496	0	0.76	RR = 0.75	0.43 ~ 1.30	0.31
Abdominal pain	8	830	832	0	0.70	RR = 0.97	0.57 ~ 1.63	0.89
Taste problem	10	690	692	0	0.46	RR = 0.10	0.05 ~ 0.17	<0.01
Constipation	5	380	378	0	0.70	RR = 0.75	0.26 ~ 2.13	0.59

#### 3.4.1 *H. pylori* eradication rate

**Figure 3:**
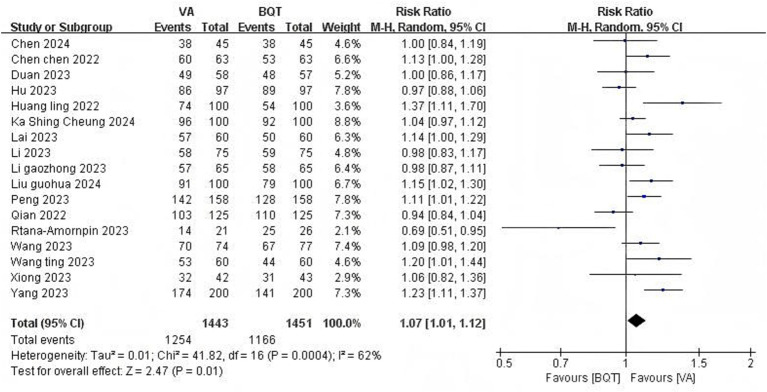
Forest plot of the meta-analysis for *H. pylori* eradication rate (ITT).

**Figure 4:**
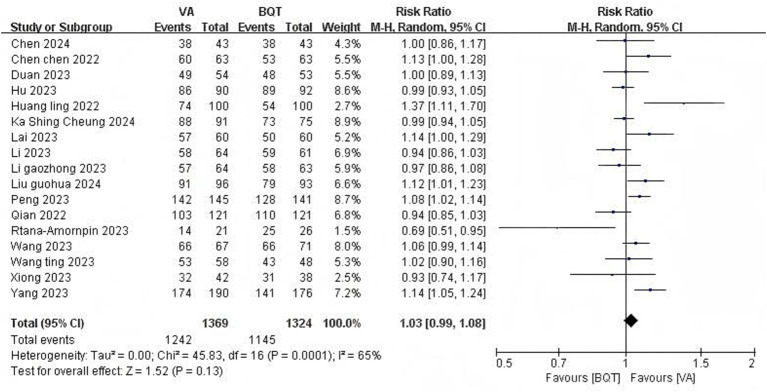
Forest plot of the meta-analysis for *H. pylori* eradication rate (PP).

All 17 RCTs (Chen et al., 2024; Chen et al., 2022; Meng et al., 2023; Hu et al., 2023; Huangling et al., 2022; Cheung Ka et al., 2024; Fujin, 2023; Li et al., 2023; Gaozhong et al., 2023; Guohua et al., 2024; Peng et al., 2023; Qian et al., 2023; Ratana-Amornpin et al., 2023; Wang et al., 2023; Ting et al., 2023; Liang et al., 2023; Yang et al., 2023) provided data of *H. pylori* eradication rate under the ITT analysis and PP analysis. There was a significant difference was observed in the eradication rate under the ITT analysis between the VA dual therapy group and BQT group (86.9% vs. 80.4%, pooled RR = 1.07, 95% CI: 1.01–1.12, *p* = 0.01) (Figure 3). High heterogeneity was observed (*I*^2^ = 62%, *P* < 0.01). There was no statistically significant in the eradication rate under the PP analysis between the VA dual therapy group and BQT group (90.7% vs. 86.5%, pooled RR = 1.03, 95% CI: 0.99–1.08, *p* = 0.13) (Figure 4). High heterogeneity was observed (*I*^2^ = 65%, *P* < 0.01).

#### 3.4.2 Overall adverse events

**Figure 5:**
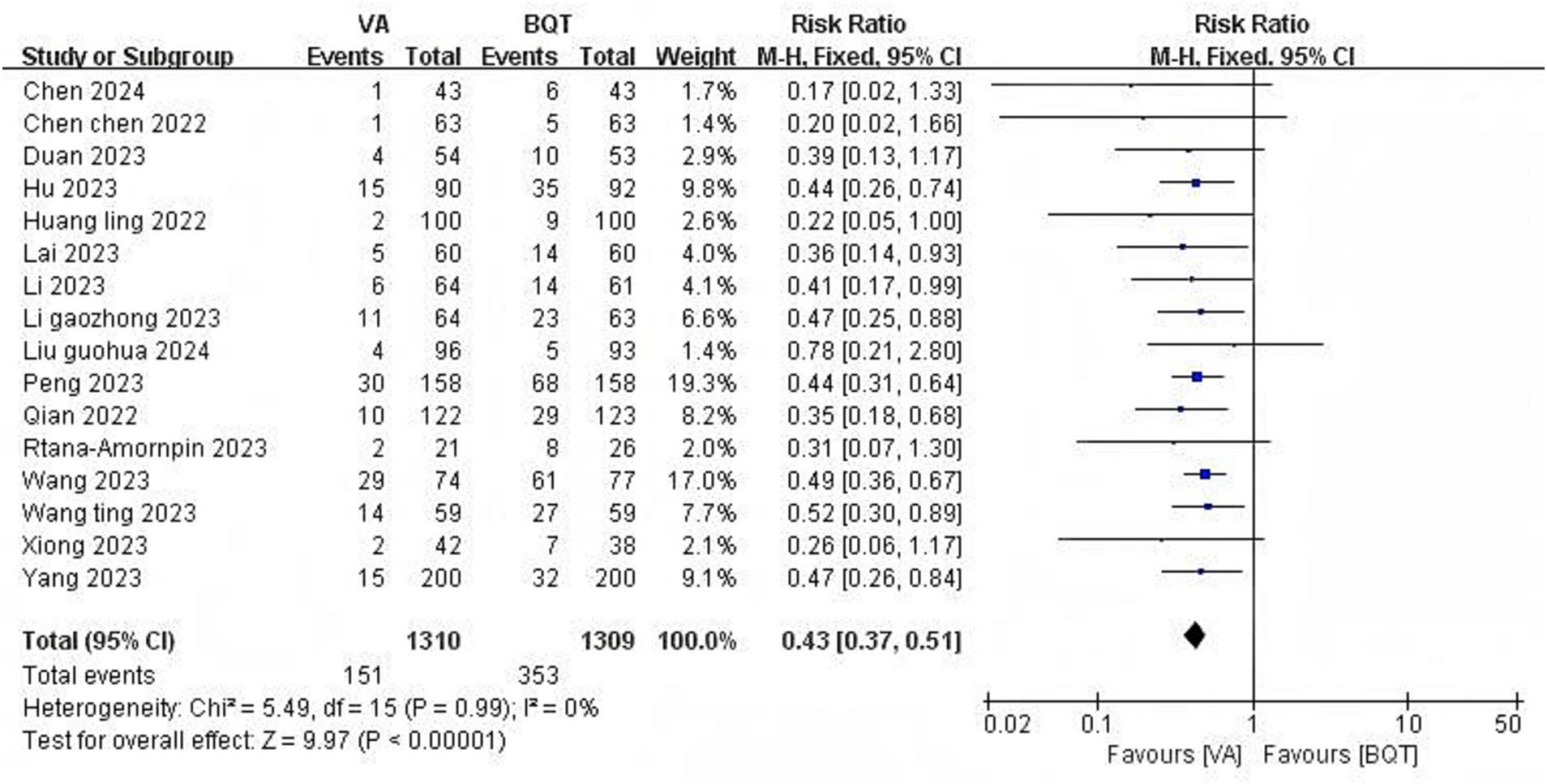
Forest plot of the meta-analysis for overall adverse events.

Totally 16 RCTs (Chen et al., 2024; Chen et al., 2022; Meng et al., 2023; Hu et al., 2023; Huangling et al., 2022; Fujin, 2023; Li et al., 2023; Gaozhong et al., 2023; Guohua et al., 2024; Peng et al., 2023; Qian et al., 2023; Ratana-Amornpin et al., 2023; Wang et al., 2023; Ting et al., 2023; Liang et al., 2023; Yang et al., 2023) involving 2,619 patients were conducted to evaluate the incidence of total adverse events. The VA dual therapy group had a significantly lower incidence of total adverse events than the BQT group (11.5% vs. 27.0%, RR = 0.43, 95% CI: 0.37–0.51, *P* < 0.01). No heterogeneity was observed (*I*^2^ = 0, *p* = 0.99) (Figure 5).

### 3.5 Secondary outcomes

#### 3.5.1 Compliance

Totally 17 RCTs (Chen et al., 2024; Chen et al., 2022; Meng et al., 2023; Hu et al., 2023; Huangling et al., 2022; Cheung Ka et al., 2024; Fujin, 2023; Li et al., 2023; Gaozhong et al., 2023; Guohua et al., 2024; Peng et al., 2023; Qian et al., 2023; Ratana-Amornpin et al., 2023; Wang et al., 2023; Ting et al., 2023; Liang et al., 2023; Yang et al., 2023) reported compliance, and the meta-analysis showed a statistically significant difference between the VA dual therapy group and BQT group (pooled RR = 1.03, 95% CI: 1.01–1.05, *p* = 0.002). There was low heterogeneity between the RCTs (*I*^2^ = 46%, *p* = 0.02) (Supplementary Figure S1).

#### 3.5.2 Nausea and/or vomiting

A total of 15 RCTs (Chen et al., 2024; Chen et al., 2022; Meng et al., 2023; Hu et al., 2023; Huangling et al., 2022; Fujin, 2023; Li et al., 2023; Gaozhong et al., 2023; Guohua et al., 2024; Peng et al., 2023; Qian et al., 2023; Ratana-Amornpin et al., 2023; Ting et al., 2023; Liang et al., 2023; Yang et al., 2023) documented the presence of nausea and/or vomiting. There is a statistically significant difference between the VA dual therapy group and BQT group, and the nausea and/or vomiting of the VA dual therapy group is lower (2.9% vs. 8.0%, RR = 0.38, 95% CI: 0.26–0.55, *P* < 0.01). There was insignificant heterogeneity between these RCTs (*I*^2^ = 4%, *p* = 0.41) (Supplementary Figure S2).

#### 3.5.3 Diarrhea

Totally 14 RCTs (Chen et al., 2022; Meng et al., 2023; Hu et al., 2023; Huangling et al., BS L., 2022; Cheung Ka et al., 2024; Fujin, 2023; Li et al., 2023; Peng et al., 2023; Qian et al., 2023; Ratana-Amornpin et al., 2023; Wang et al., 2023; Ting et al., 2023; Liang et al., 2023; Yang et al., 2023) reported the existence of diarrhea in the VA dual therapy group and the BQT group and three studies (Chen et al., 2024; Gaozhong et al., 2023; Guohua et al., 2024) did not report it. The aggregated findings demonstrated that there was no statistically significant disparity between two groups, and indicate insignificant heterogeneity was observed (RR = 0.75, 95%CI: 0.55–1.03, *p* = 0.08; *I*^2^ = 6%, *p* = 0.39) (Supplementary Figure S3).

#### 3.5.4 Skin rash

There were a total of 10 RCTs (Hu et al., 2023; Huangling et al., 2022; Fujin, 2023; Li et al., 2023; Gaozhong et al., 2023; Peng et al., 2023; Qian et al., 2023; Wang et al., 2023; Ting et al., 2023; Liang et al., 2023) that provided evidence for the presence of skin rash. The aggregated findings suggested that there was no statistically significant disparity and no heterogeneity between two groups (RR = 0.76, 95%CI: 0.42–1.38, *p* = 0.36; *I*^2^ = 0, *p* = 0.64) (Supplementary Figure S4).

#### 3.5.5 Dizziness and/or headaches

There were seven RCTs (Chen et al., 2024; Hu et al., 2023; Huangling et al., 2022; Li et al., 2023; Peng et al., 2023; Qian et al., 2023; Yang et al., 2023) that confirmed the existence of dizziness and/or headaches. The combined results showed that the occurrence rate of dizziness and/or headaches was markedly greater in the BQT group compared to the VA dual therapy group (0.6% vs. 2.2%, RR = 0.33, 95%CI: 0.13–0.84, *p* = 0.02; *I*^2^ = 0, *p* = 0.65) (Supplementary Figure S5).

#### 3.5.6 Abdominal distension

Abdominal distension of two groups was reported in seven RCTs (Chen et al., 2024; Meng et al., 2023; Hu et al., 2023; Cheung Ka et al., 2024; Qian et al., 2023; Ratana-Amornpin et al., 2023; Ting et al., 2023). There was no statistically significant difference between two groups (RR = 0.75, 95%CI: 0.43–1.30, *p* = 0.31). No heterogeneity was observed (*I*^2^ = 0, *p* = 0.76) (Supplementary Figure S6).

#### 3.5.7 Abdominal pain

Eight RCTs (Chen et al., 2024; Chen et al., 2022; Meng et al., 2023; Hu et al., 2023; Cheung Ka et al., 2024; Peng et al., 2023; Qian et al., 2023; Yang et al., 2023) reported the existence of abdominal pain between the VA dual therapy group and the BQT group. The aggregated findings demonstrated that there was no statistically significant disparity between two groups (RR = 0.97, 95%CI: 0.57–1.63, *p* = 0.89). No heterogeneity was observed (*I*^2^ = 0, *p* = 0.70) (Supplementary Figure S7).

#### 3.5.8 Taste problem

A total of 10 RCTs (Meng et al., 2023; Hu et al., 2023; Cheung Ka et al., 2024; Li et al., 2023; Gaozhong et al., 2023; Qian et al., 2023; Ratana-Amornpin et al., 2023; Wang et al., 2023; Ting et al., 2023; Liang et al., 2023) documented the presence of taste problem. There is a statistically significant difference between the VA dual therapy group and BQT group, and the incidence of taste problem in the VA dual therapy group is lower (1.3% vs. 17.3%, RR = 0.10, 95%CI: 0.05–0.17, *P* < 0.01). No heterogeneity was observed (*I*^2^ = 0, *p* = 0.46) (Supplementary Figure S8).

#### 3.5.9 Constipation

There were a total of five RCTs (Chen et al., 2024; Fujin, 2023; Guohua et al., 2024; Qian et al., 2023; Ting et al., 2023) that provided evidence for the presence of constipation. The aggregated findings suggested that there was no statistically significant disparity between the two groups (RR = 0.75, 95%CI: 0.26–2.13, *p* = 0.59). There was no heterogeneity (*I*^2^ = 0, *p* = 0.70) (Supplementary Figure S9).

#### 3.5.10 Cost

Four RCTs (Chen et al., 2024; Li et al., 2023; Liang et al., 2023; Yang et al., 2023) documented the expenses associated with the VA dual therapy group and the BQT group. The calculations indicated that, in comparison to the BQT group, the VA group across all four categories exhibited reduced costs and cost-effectiveness ratios. Table 3 presented and contrasted the information.

**Table 3:** Cost–benefit of therapy in some included studies.

Study	Country	Cost		Eradication rate		Cost-effectiveness ratio	
		VA (RMB)	BQT (RMB)	VA	BQT	VA	BQT
Chen et al. (2024)	China	57.46	70.27	38/45	38/45	0.68	0.83
Li et al. (2023)	China	285.06	317.19	58/75	59/75	3.69	4.03
Liang et al. (2023)	China	180	190	35/42	31/43	2.16	2.64
Yang et al. (2023)	China	226.39	447.32	174/200	141/200	2.60	6.34

### 3.6 Publication bias

Funnel plots were used to evaluate publication bias. The bilaterally symmetrical funnel plot did not provide any apparent indications of publication bias with regards to *H. pylori* eradication rate under the ITT analysis (Supplementary Figure S10) or PP analysis (Supplementary Figure S11) or overall adverse events (Supplementary Figure S12).

## 4 Discussion

The objective of the meta-analysis was to compare the effectiveness, safety, compliance and cost-effectiveness of VA dual therapy against BQT in eliminating *H. pylori*. The results indicated notable disparities in the *H. pylori* eradication rate, total adverse events, and compliance between the two groups, with VA dual therapy being more effective than BQT. Furthermore, VA dual therapy has lower cost compared to BQT. Regarding adverse events, VA significantly decreased the incidence of nausea, vomiting, dizziness, headache, and taste problem compared with BQT. However, there were no statistically significant differences in the incidence of diarrhea, abdominal pain, skin rash, abdominal discomfort, and constipation.

The treatment of *H. pylori*, responsible for conditions like gastritis and gastric cancer, depends heavily on antibiotics. Effective eradication requires antibiotic use, often combined with PPIs to optimize the PH-dependent action of these drugs (Yokota et al., 2022; Kato et al., 2019; Buzás and Newer, 2023). Standard triple therapy (STT) consisting of a PPI, amoxicillin, and clarithromycin has been the primary treatment (Shin et al., 2021). PPIs, which suppress stomach acid secretion by selectively inhibiting H+/K+-ATPase in parietal cells, undergo metabolism by the CYP2C19 enzyme (Aumpan et al., 2022). Nevertheless, the limited duration of their action and the diverse pharmacokinetics among various populations restrict their efficacy in eliminating *H. pylori* (Strand et al., 2017). Molecular changes in the 23S rRNA gene can diminish the binding affinity of clarithromycin, which specifically binds to the 23S rRNA of the bacterial ribosome (Erah et al., 1997). The inappropriate utilization of current treatment regimens, in conjunction with the broad-spectrum efficacy of current medication, leads to higher frequencies of antibiotic resistance in both *H. pylori* (Vita et al., 2022). So, for the treatment of gastroduodenal ulcers and *H. pylori* infection, bismuth-based therapies such as colloidal bismuth subcitrate and other salts are employed (Alkim et al., 2017). A comprehensive study of 3,990 patients revealed that treatment regimens using bismuth were more effective than regimens without bismuth, resulting in an eradication rate of 85.8% compared to 72.9% (Ko et al., 2019). This improvement was particularly significant in strains that were resistant to clarithromycin and levofloxacin (Ko et al., 2019). Monotherapy with bismuth alone effectively eliminated *H. pylori* in 20% of cases, and the addition of bismuth to triple therapy increased eradication rates by 30–40% in strains that were resistant (Hu et al., 2022; Hu et al., 2020). The antibacterial activity of bismuth against *H. pylori* is attributed to its multitarget interactions, which involve the inhibition of metallo-β-lactamases and bacterial enzymes, particularly MBLs containing cysteine residues (Li et al., 2018). Bismuth confers direct bactericidal effects by the formation of complexes in the bacterial wall, which hinders ATP generation and prevents bacterial attachment to the stomach mucosa (Strand et al., 2017). The method of action of this substance involves the suppression of protein synthesis, membrane function, and regulation of oxidative stress, among other effects (Luther et al., 2010; Rawla et al., 2018; Oshima and Miwa, 2018; Kagami et al., 2016). Owing to these several processes, bismuth is extensively employed in therapeutic practice. A meta-analysis of nine randomized controlled trials (*N* = 1,679) revealed that BQT achieved an eradication rate of 78.3%, which was similar to clarithromycin triple therapy at 77%, without any notable disparity in adverse effects (Luther et al., 2010). Quadruple effect is better, so it is more commonly used clinically.

Our results further indicated that the efficacy of vonoprazan-based dual therapy is superior to bismuth quadruple therapy. P-CABs are newly developed pharmaceuticals that selectively sequester potassium ions and inhibit the H+, K+ ATPase enzyme, therefore inhibiting the synthesis of acids, which are characterized by their direct binding to the enzyme with a rapid action, without requiring acid activation (Rawla et al., 2018). Its mechanism of action differs from that of typical PPIs, which has two advantages compared to conventional PPIs: it becomes completely active on the first day and provides strong acid suppression (Oshima and Miwa, 2018). What’s more, vonoprazan exhibits stability under acidic conditions and is not influenced by CYP2C19 genotypes or food consumption (Oshima and Miwa, 2018), ensuring constant effectiveness in various patient groups (Kagami et al., 2016), and demonstrating the highest potency, even at a pH of 6.5 (Hori et al., 2010). Amoxicillin, a commonly employed antibiotic for eliminating *H. pylori*, interferes with the assembly of peptidoglycan and impairs the cell walls of bacteria by attaching to penicillin-binding proteins (PBPs) (Wang et al., 2023). In combination with amoxicillin, vonoprazan consistently maintains intragastric pH levels above 5, making it effective in preventing recurrent bleeding after endoscopic hemostasis and as a crucial agent in *H. pylori* eradication therapy (Graham and Dore, 2018). Vonoprazan exhibits strong affinity for the ion-binding site of H+/K+-ATPase, rendering it very efficient in acidic settings. Its pKa value of 9.37 guarantees swift protonation and strong inhibitory effects, even in neutral conditions (Hori et al., 2010). Due to its slow dissociation rate from H+/K+-ATPase and resistance to degradation in acidic conditions, the effectiveness of VA dual therapy is enhanced when combined with amoxicillin, resulting in high eradication rates (Oshima and Miwa, 2018).

The results of our study indicated that BQT treatment resulted in a greater number of adverse events in comparison to VA dual therapy. Specifically, there were increased occurrences of nausea, vomiting, dizziness, headache, and taste problem. Gastrointestinal irritation resulting from drug absorption might result in symptoms such as nausea, vomiting, abdominal distension, and intestinal diarrhea. This phenomenon arises from direct harm to the gastric mucosa, compromised gastrointestinal peristalsis, and changes in nerve conduction and blood circulation, leading to the enlargement of blood vessels and irritation. Although often minor and temporary in adverse effects, bismuth compounds can induce dark stools, slight dizziness, headache, and diarrhea (Strand et al., 2017). Prevalent adverse effects include taste problem and black stool, which, although reversible, can impact patient adherence and diminish the effectiveness of treatment (Wang et al., 2023). Furthermore, in comparison to the VA group, the BQT group has a larger cost and cost-effectiveness ratio, indicating that it incurs greater expenses to attain same outcomes, hence diminishing patient compliance. The intricacy of drug administration in BQT further reduces adherence, which subsequently affects the outcome of treatment (Hu et al., 2022). Bismuth salts employed in medicine exhibit low solubility in water, resulting in limited absorption efficiency. The majority of bismuth consumed is eliminated as bismuth sulfide, resulting in the production of black stools, whereas only a restricted quantity is absorbed and eliminated in the urine (Alkim et al., 2017). The hydrolysis-induced production of Bi38 clusters is a contributing factor to the limited solubility of bismuth subsalicylate in freshwater solutions (Ge and Sun, 2007). The predominant adverse effects of PPIs encompass abdominal pain, diarrhea, constipation, nausea, and vomiting, but these adverse effects are manageable and diminish once the medicine is stopped (Yu et al., 2017). PPIs reduce stomach acid output by inhibiting the H+/K+ ATPase enzyme in parietal cells, which is the last stage of acid clearance (El Rouby et al., 2018). Yet, decreased gastric acid might hinder the process of digestion, resulting in symptoms such as abdominal distension and irregular bowel movements. Omeprazole has been shown to have the function of delaying gastric emptying compared to placebo, either acid inhibition leads to an increase in gastrin levels, which delays gastric emptying, or it is possible that decreased acid secretion reduces the activation of digestive enzymes such as pepsin or the amount of fluid in the stomach, which leads to an increase in viscosity (Gerson et al., 2011). This may be the cause of bloating. A nocturnal acid breakthrough (NAB), characterized by increased nighttime intragastric acidity, frequently arises even with conventional PPI treatment, resulting in symptoms including acid reflux and taste problem in the mouth (Scarpignato et al., 2016). Both antibiotics and PPIs used to eliminate *H. pylori* can induce substantial changes in the gut microbiota, leading to a decrease in microbial diversity and an elevated susceptibility to gut infections (Wang et al., 2024). Dysbiosis of gut microbiota can cause relaxation of intestinal muscles, affect gastrointestinal motility, and lead to symptoms such as constipation and diarrhea. VA dual therapy did not decrease alpha diversity or gut microbiota abundance, nor did it alter beta diversity, and had a minimal impact on gut microbiota diversity and relative abundance (Horii et al., 2021). The skin rash may be related to allergic reactions to medication. Amoxicillin, used in eradication regimens, can cause hypersensitivity reactions: type-I IgE can cause urticarial rashes or severe anaphylaxis, whereas type-IV can cause a non-itchy, papular, or morbilliform rash (Torres and Blanca, 2010).

Based on our current understanding, this meta-analysis incorporates the latest study that compares VA dual therapy and BQT in the treatment of *H. pylori* infection. We have included a substantial number of studies compared to earlier publications, which may result in a more reliable conclusion. Our findings offer significant insights into the management of *H. pylori* infection and make a vital contribution to both clinical practice and research in this field. However, our study is not without its limitations. First, regarding the inclusion of patients with *H. pylori*, we did not identify patients who had received treatment for the first time. Instead, we focused on patients who were infected and had not been cured. We did not impose any limitations on patient characteristics such as age, race, and gender, which could result in heterogeneity and bias. We failed to conduct a subgroup analysis regarding pediatric patients and adult patients, since the original literature did not provide data for separate population. Besides, most of the studies included were conducted in China. This narrow focus limits the generalizability of the findings to the broader population. Clinical trials assessing the efficacy and safety of vonoprazan or other potassium-competitive acid blockers for patients with *Helicobacter pylori* infection have also been conducted in other countries, including Pakistan, South Korea, Japan, etc. (Horii et al., 2021; Zuberi et al., 2022; Chey et al., 2022; Gotoda et al., 2020; Suzuki et al., 2020; Furuta et al., 2020; Nizam et al., 2024; Huh et al., 2021; Oh et al., 2023; Kim et al., 2023; Jung et al., 2024; Cho and Jin, 2024; Tungtrongchitr et al., 2024; Farzana et al., 2024; Maruyama et al., 2017). For example, a trial conducted in Pakistan compared VA versus standard triple therapy with PPI (Amoxicillin + Clarithromycin + Omeprazole) (Horii et al., 2021), while five trials conducted in Japan compared VA versus vonoprazan triple therapy (Zuberi et al., 2022; Chey et al., 2022; Gotoda et al., 2020; Suzuki et al., 2020; Furuta et al., 2020). Four trials conducted in Pakistan or South Korea compared BQT based on potassium-competitive acid blockers versus BQT based on PPI (Nizam et al., 2024; Huh et al., 2021; Oh et al., 2023; Kim et al., 2023). However, this meta-analysis was design to compare the efficacy and safety of vonoprazan-amoxicillin dual therapy in comparison to bismuth-containing quadruple therapy, and these trials (Horii et al., 2021; Zuberi et al., 2022; Chey et al., 2022; Gotoda et al., 2020; Suzuki et al., 2020; Furuta et al., 2020; Nizam et al., 2024; Huh et al., 2021; Oh et al., 2023; Kim et al., 2023; Jung et al., 2024; Cho and Jin, 2024; Tungtrongchitr et al., 2024; Farzana et al., 2024; Maruyama et al., 2017) were excluded since the regimens of the intervention group or the control group did not meet our inclusion criteria. It is advised to perform RCTs comparing VA versus BQT in different regions, which could help mitigate selection bias and strengthen the conclusions.

In conclusion, this study demonstrated that VA dual therapy exhibited a superior eradication rate, increased adherence, reduced unpleasant effects, and decreased cost in comparison to BQT for treatment-naive patients with *H. pylori* infection.

## Data Availability

The datasets presented in this study can be found in online repositories. The names of the repository/repositories and accession number(s) can be found in the article/Supplementary material.
