# Supplementary material for: Efficacy and safety of vonoprazan-amoxicillin dual therapy versus bismuth-containing quadruple therapy for patients with Helicobacter pylori infection: a meta-analysis
Source: Front Microbiol. 2025 Mar 19;16:1561749. doi: 10.3389/fmicb.2025.1561749 (PMC11962034; doi:10.3389/fmicb.2025.1561749)

Figure S1. Forest plot of the meta-analysis for compliance

Study or Subgroup	VA Events	VA Total	BQT Events	BQT Total	Weight	Risk Ratio M-H, Fixed, 95% CI
Chen 2024	43	45	43	45	3.2%	1.00 [0.91, 1.09]
Chen chen 2022	63	63	63	63	4.8%	1.00 [0.97, 1.03]
Duan 2023	54	58	53	57	4.0%	1.00 [0.91, 1.11]
Hu 2023	90	97	92	97	6.9%	0.98 [0.91, 1.05]
Huang ling 2022	100	100	100	100	7.5%	1.00 [0.98, 1.02]
Ka Shing Cheung 2024	95	100	88	100	6.6%	1.08 [0.99, 1.18]
Lai 2023	60	60	60	60	4.5%	1.00 [0.97, 1.03]
Li 2023	64	75	61	75	4.6%	1.05 [0.91, 1.21]
Li gaozhong 2023	64	65	63	65	4.7%	1.02 [0.96, 1.07]
Liu guohua 2024	96	100	93	100	7.0%	1.03 [0.97, 1.10]
Peng 2023	144	158	141	158	10.6%	1.02 [0.95, 1.10]
Qian 2022	121	122	120	123	9.0%	1.02 [0.98, 1.05]
Rtana-Amornpin 2023	21	21	26	26	1.8%	1.00 [0.92, 1.09]
Wang 2023	67	74	71	77	5.2%	0.98 [0.89, 1.08]
Wang ting 2023	58	60	50	60	3.8%	1.16 [1.03, 1.31]
Xiong 2023	39	42	32	38	2.5%	1.10 [0.94, 1.30]
Yang 2023	190	200	176	200	13.2%	1.08 [1.02, 1.15]
**Total (95% CI)**		**1440**		**1444**	**100.0%**	**1.03 [1.01, 1.05]**
Total events	1369		1332			

Heterogeneity: Chi² = 29.68, df = 16 (P = 0.02); I² = 46%
Test for overall effect: Z = 3.15 (P = 0.002)

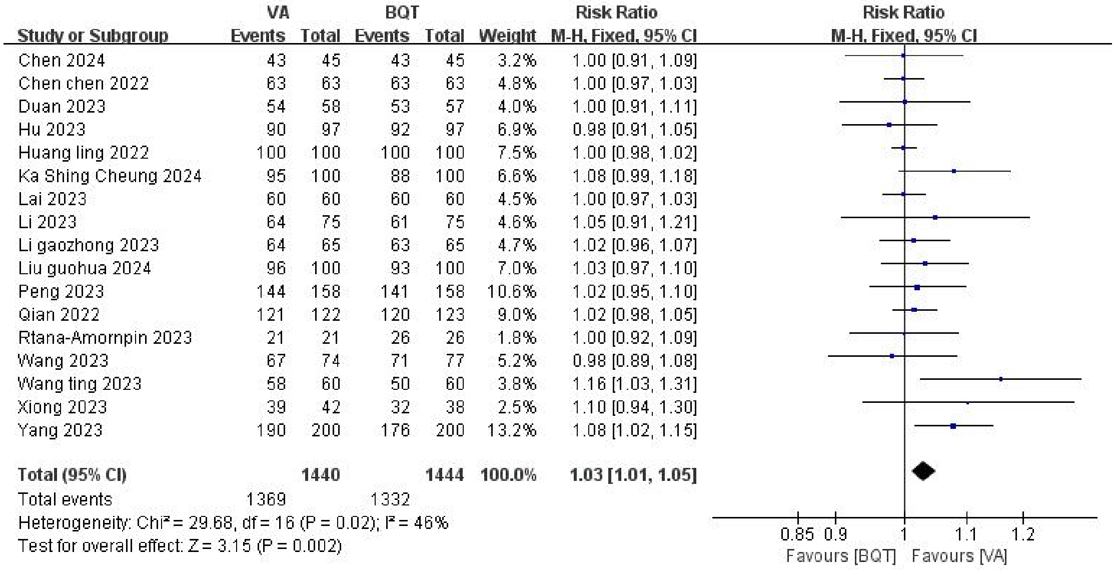

Figure S2. Forest plot of the meta-analysis for nausea and(or) vomiting

Study or Subgroup	VA Events	VA Total	BQT Events	BQT Total	Weight	Risk Ratio M-H, Fixed, 95% CI
Chen 2024	1	43	1	43	1.0%	1.00 [0.06, 15.48]
Chen chen 2022	0	63	2	63	2.5%	0.20 [0.01, 4.08]
Duan 2023	2	54	1	53	1.0%	1.96 [0.18, 21.01]
Hu 2023	1	90	15	92	14.8%	0.07 [0.01, 0.51]
Huang ling 2022	1	100	4	100	4.0%	0.25 [0.03, 2.20]
Lai 2023	1	60	0	60	0.5%	3.00 [0.12, 72.20]
Li 2023	4	64	4	61	4.1%	0.95 [0.25, 3.64]
Li gaozhong 2023	3	64	13	63	13.1%	0.23 [0.07, 0.76]
Liu guohua 2024	3	96	2	93	2.0%	1.45 [0.25, 8.50]
Peng 2023	10	158	22	158	22.0%	0.45 [0.22, 0.93]
Qian 2022	3	122	4	123	4.0%	0.76 [0.17, 3.31]
Rtana-Amornpin 2023	0	21	2	26	2.2%	0.25 [0.01, 4.85]
Wang ting 2023	2	59	4	59	4.0%	0.50 [0.10, 2.63]
Xiong 2023	0	42	3	38	3.7%	0.13 [0.01, 2.43]
Yang 2023	5	200	21	200	21.0%	0.24 [0.09, 0.62]
**Total (95% CI)**		**1236**		**1232**	**100.0%**	**0.38 [0.26, 0.55]**
Total events	36		98			

Heterogeneity: Chi² = 14.53, df = 14 (P = 0.41); I² = 4%
Test for overall effect: Z = 5.22 (P < 0.00001)

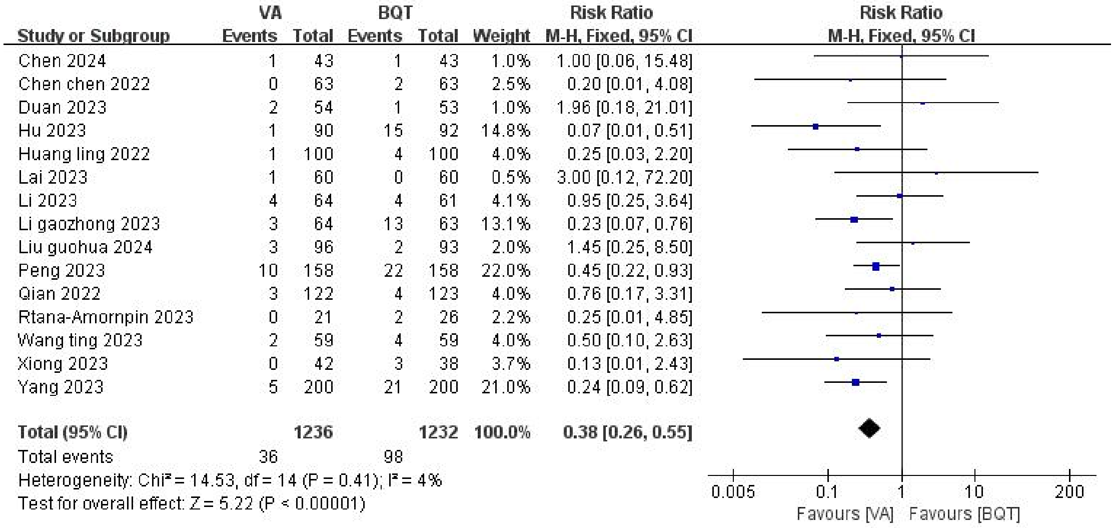

Figure S3. Forest plot of the meta-analysis for diarrhea

Study or Subgroup	VA Events	VA Total	BQT Events	BQT Total	Weight	Risk Ratio M-H, Fixed, 95% CI
Chen chen 2022	0	63	1	63	1.8%	0.33 [0.01, 8.03]
Duan 2023	0	54	4	53	5.5%	0.11 [0.01, 1.98]
Hu 2023	4	90	0	92	0.6%	9.20 [0.50, 168.41]
Huang ling 2022	0	100	2	100	3.0%	0.20 [0.01, 4.11]
Ka Shing Cheung 2024	12	100	9	100	10.9%	1.33 [0.59, 3.02]
Lai 2023	2	60	9	60	10.9%	0.22 [0.05, 0.99]
Li 2023	0	64	2	61	3.1%	0.19 [0.01, 3.89]
Peng 2023	15	158	16	158	19.4%	0.94 [0.48, 1.83]
Qian 2022	3	122	6	123	7.2%	0.50 [0.13, 1.97]
Rtana-Amornpin 2023	1	21	1	26	1.1%	1.24 [0.08, 18.64]
Wang 2023	15	74	17	77	20.2%	0.92 [0.50, 1.70]
Wang ting 2023	4	59	9	59	10.9%	0.44 [0.14, 1.36]
Xiong 2023	1	42	0	38	0.6%	2.72 [0.11, 64.85]
Yang 2023	2	200	4	200	4.8%	0.50 [0.09, 2.70]
**Total (95% CI)**		**1207**		**1210**	**100.0%**	**0.75 [0.55, 1.03]**
Total events	59		80			

Heterogeneity: Chi² = 13.76, df = 13 (P = 0.39); I² = 6%

Test for overall effect: Z = 1.77 (P = 0.08)

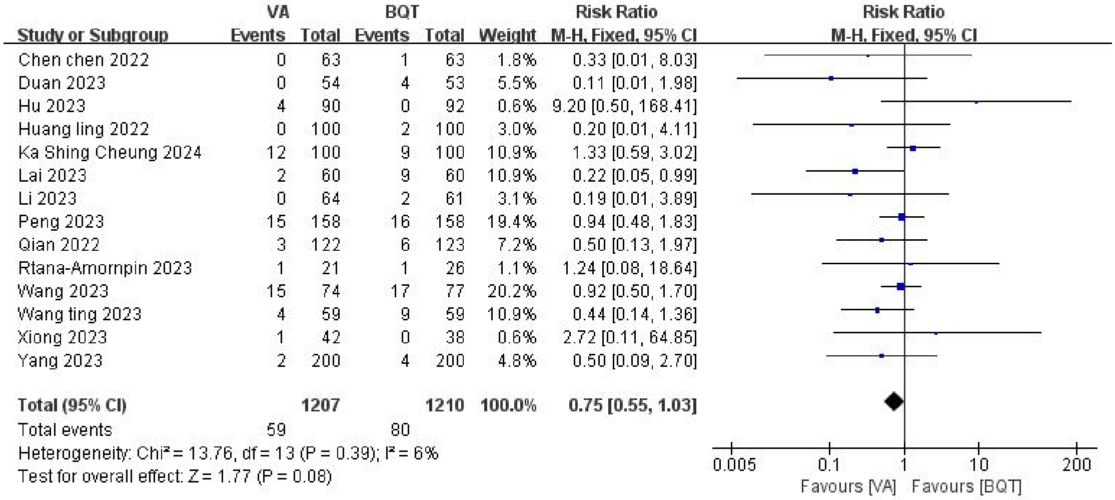

Figure S4. Forest plot of the meta-analysis for skin rash

Study or Subgroup	VA Events	VA Total	BQT Events	BQT Total	Weight	Risk Ratio M-H, Fixed, 95% CI
Hu 2023	2	90	0	92	2.0%	5.11 [0.25, 104.98]
Huang ling 2022	1	100	1	100	4.1%	1.00 [0.06, 15.77]
Lai 2023	1	60	3	60	12.3%	0.33 [0.04, 3.11]
Li 2023	0	64	1	61	6.3%	0.32 [0.01, 7.66]
Li gaozhong 2023	1	64	0	63	2.1%	2.95 [0.12, 71.17]
Peng 2023	2	158	7	158	28.7%	0.29 [0.06, 1.35]
Qian 2022	3	122	1	123	4.1%	3.02 [0.32, 28.68]
Wang 2023	5	74	8	77	32.1%	0.65 [0.22, 1.90]
Wang ting 2023	0	59	1	59	6.1%	0.33 [0.01, 8.02]
Xiong 2023	1	42	0	38	2.1%	2.72 [0.11, 64.85]
**Total (95% CI)**		**833**		**831**	**100.0%**	**0.76 [0.42, 1.38]**
Total events	16		22			

Heterogeneity: Chi² = 7.00, df = 9 (P = 0.64); I² = 0%

Test for overall effect: Z = 0.91 (P = 0.36)

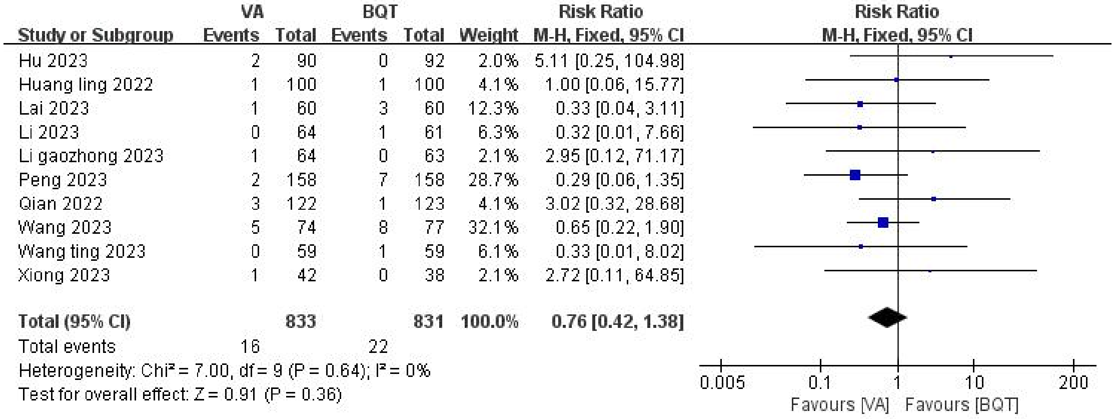

Figure S5. Forest plot of the meta-analysis for dizziness and(or)headaches

Study or Subgroup	VA Events	VA Total	BQT Events	BQT Total	Weight	Risk Ratio M-H, Fixed, 95% CI
Chen 2024	1	43	1	43	5.6%	1.00 [0.06, 15.48]
Hu 2023	0	90	9	92	52.5%	0.05 [0.00, 0.91]
Huang ling 2022	0	100	2	100	14.0%	0.20 [0.01, 4.11]
Li 2023	1	64	1	61	5.7%	0.95 [0.06, 14.90]
Peng 2023	1	158	1	158	5.6%	1.00 [0.06, 15.85]
Qian 2022	1	122	2	123	11.1%	0.50 [0.05, 5.49]
Yang 2023	1	200	1	200	5.6%	1.00 [0.06, 15.88]
**Total (95% CI)**		**777**		**777**	**100.0%**	**0.33 [0.13, 0.84]**
Total events	5		17			

Heterogeneity: Chi² = 4.21, df = 6 (P = 0.65); I² = 0%
Test for overall effect: Z = 2.33 (P = 0.02)

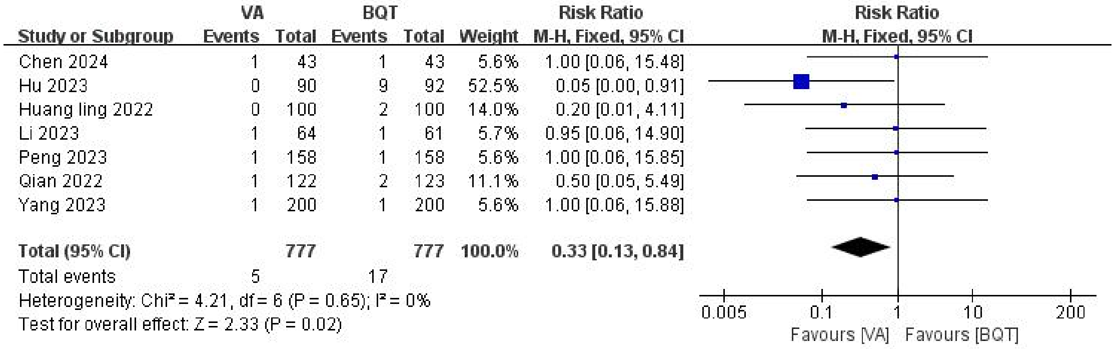

Figure S6. Forest plot of the meta-analysis for abdominal distension

Study or Subgroup	VA Events	VA Total	BQT Events	BQT Total	Weight	Risk Ratio M-H, Fixed, 95% CI
Chen 2024	0	43	1	43	5.6%	0.33 [0.01, 7.96]
Duan 2023	1	54	2	53	7.5%	0.49 [0.05, 5.25]
Hu 2023	3	90	0	92	1.8%	7.15 [0.37, 136.55]
Ka Shing Cheung 2024	10	100	14	100	52.1%	0.71 [0.33, 1.53]
Qian 2022	1	122	3	123	11.1%	0.34 [0.04, 3.19]
Rtana-Amornpin 2023	1	21	1	26	3.3%	1.24 [0.08, 18.64]
Wang ting 2023	3	59	5	59	18.6%	0.60 [0.15, 2.40]
**Total (95% CI)**		**489**		**496**	**100.0%**	**0.75 [0.43, 1.30]**
Total events	19		26			

Heterogeneity: Chi² = 3.35, df = 6 (P = 0.76); I² = 0%
Test for overall effect: Z = 1.02 (P = 0.31)

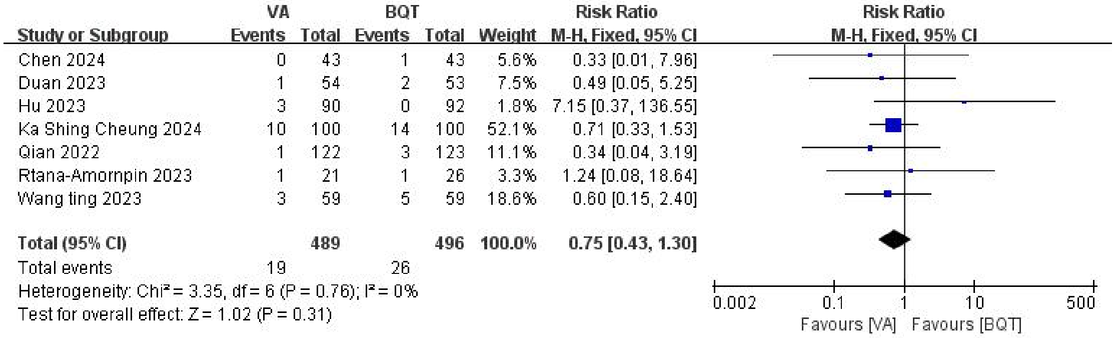

Figure S7. Forest plot of the meta-analysis for abdominal pain

Study or Subgroup	VA Events	VA Total	BQT Events	BQT Total	Weight	Risk Ratio M-H, Fixed, 95% CI
Chen 2024	0	43	3	43	12.7%	0.14 [0.01, 2.68]
Chen chen 2022	1	63	0	63	1.8%	3.00 [0.12, 72.27]
Duan 2023	1	54	0	53	1.8%	2.95 [0.12, 70.72]
Hu 2023	1	90	3	92	10.8%	0.34 [0.04, 3.21]
Ka Shing Cheung 2024	8	100	6	100	21.9%	1.33 [0.48, 3.70]
Peng 2023	8	158	9	158	32.8%	0.89 [0.35, 2.25]
Qian 2022	2	122	3	123	10.9%	0.67 [0.11, 3.95]
Yang 2023	4	200	2	200	7.3%	2.00 [0.37, 10.80]
**Total (95% CI)**		**830**		**832**	**100.0%**	**0.97 [0.57, 1.63]**
Total events	25		26			

Heterogeneity: Chi² = 4.71, df = 7 (P = 0.70); I² = 0%
Test for overall effect: Z = 0.13 (P = 0.89)

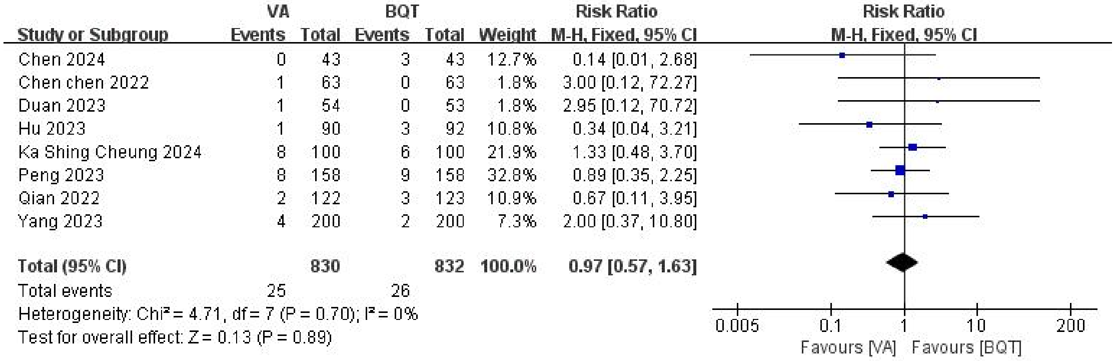

Figure S8. Forest plot of the meta-analysis for taste problem

Study or Subgroup	VA Events	VA Total	BQT Events	BQT Total	Weight	Risk Ratio M-H, Fixed, 95% CI
Duan 2023	0	54	1	53	1.3%	0.33 [0.01, 7.86]
Hu 2023	2	90	4	92	3.3%	0.51 [0.10, 2.72]
Ka Shing Cheung 2024	2	100	18	100	14.9%	0.11 [0.03, 0.47]
Li 2023	0	64	1	61	1.3%	0.32 [0.01, 7.66]
Li gaozhong 2023	2	64	9	63	7.5%	0.22 [0.05, 0.97]
Qian 2022	0	122	19	123	16.1%	0.03 [0.00, 0.42]
Rtana-Amornpin 2023	0	21	6	26	4.8%	0.09 [0.01, 1.59]
Wang 2023	2	74	52	77	42.1%	0.04 [0.01, 0.16]
Wang ting 2023	1	59	7	59	5.8%	0.14 [0.02, 1.13]
Xiong 2023	0	42	3	38	3.0%	0.13 [0.01, 2.43]
**Total (95% CI)**		**690**		**692**	**100.0%**	**0.10 [0.05, 0.17]**
Total events	9		120			

Heterogeneity: Chi² = 8.78, df = 9 (P = 0.46); I² = 0%
Test for overall effect: Z = 7.73 (P < 0.00001)

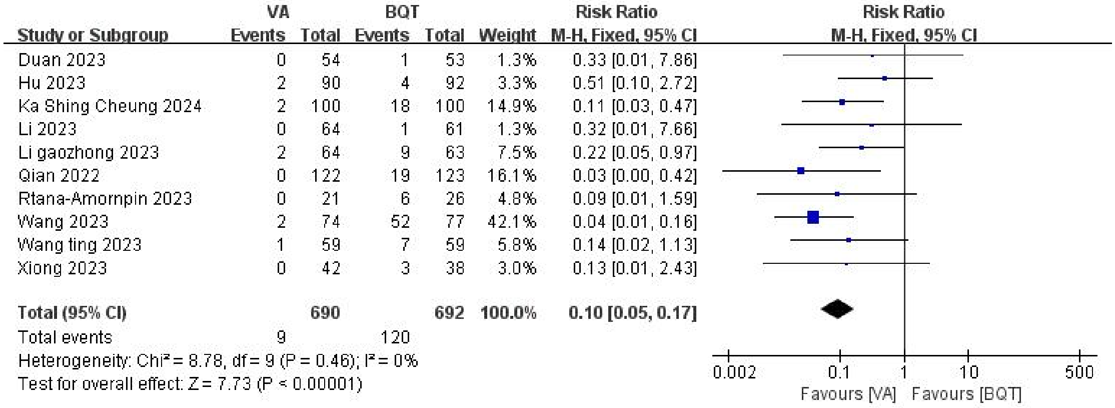

Figure S9. Forest plot of the meta-analysis for constipation

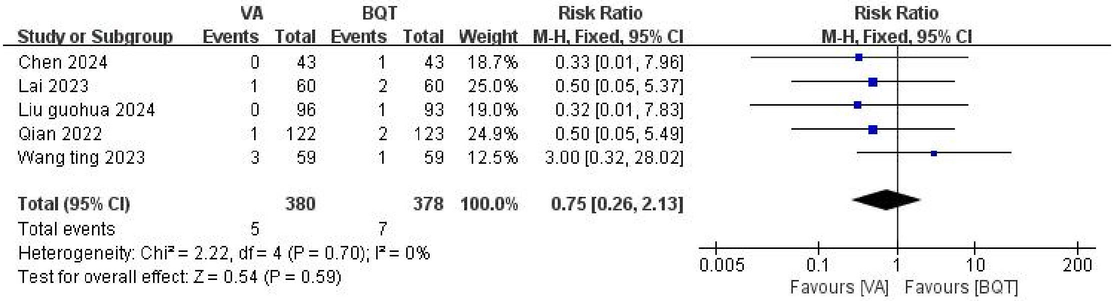

Figure S10. Funnel plot of the meta-analysis for H.pylori eradication rate(ITT)

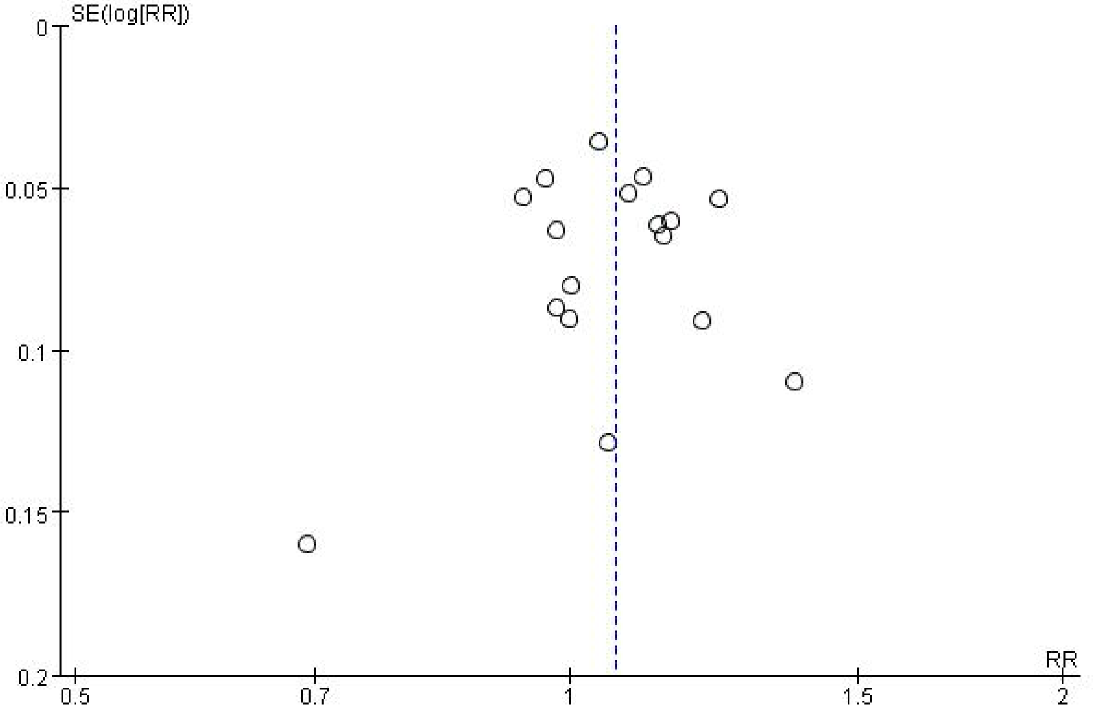

Figure S11. Funnel plot of the meta-analysis for H.pylori eradication rate(PP)

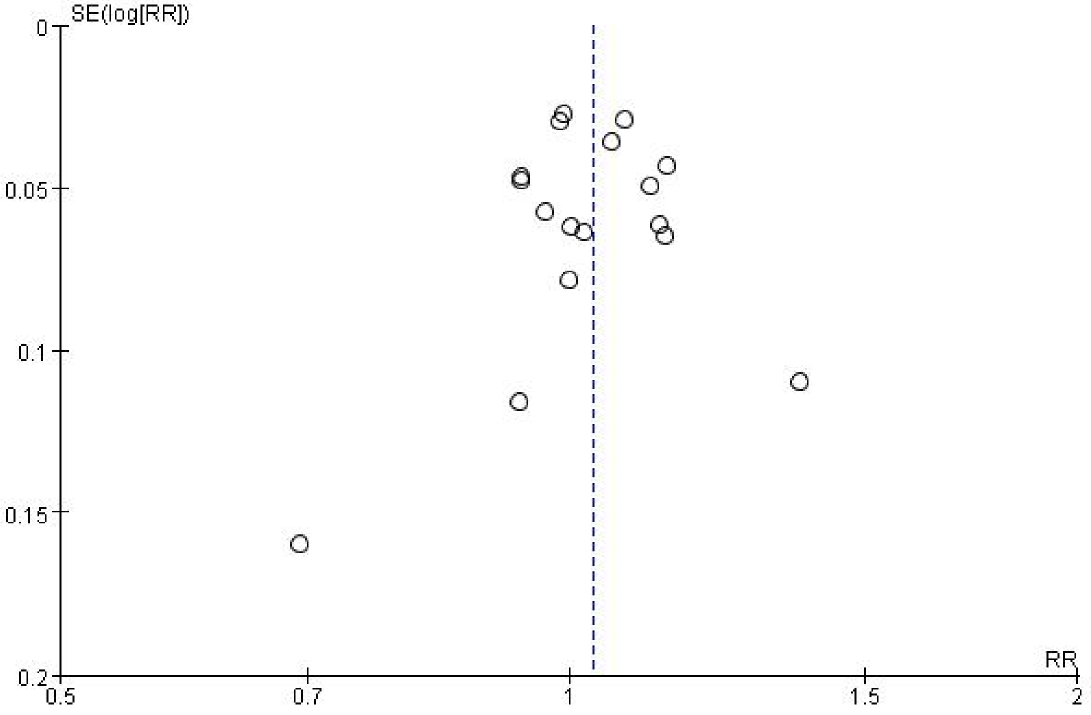

Figure S12. Funnel plot of the meta-analysis for overall adverse events

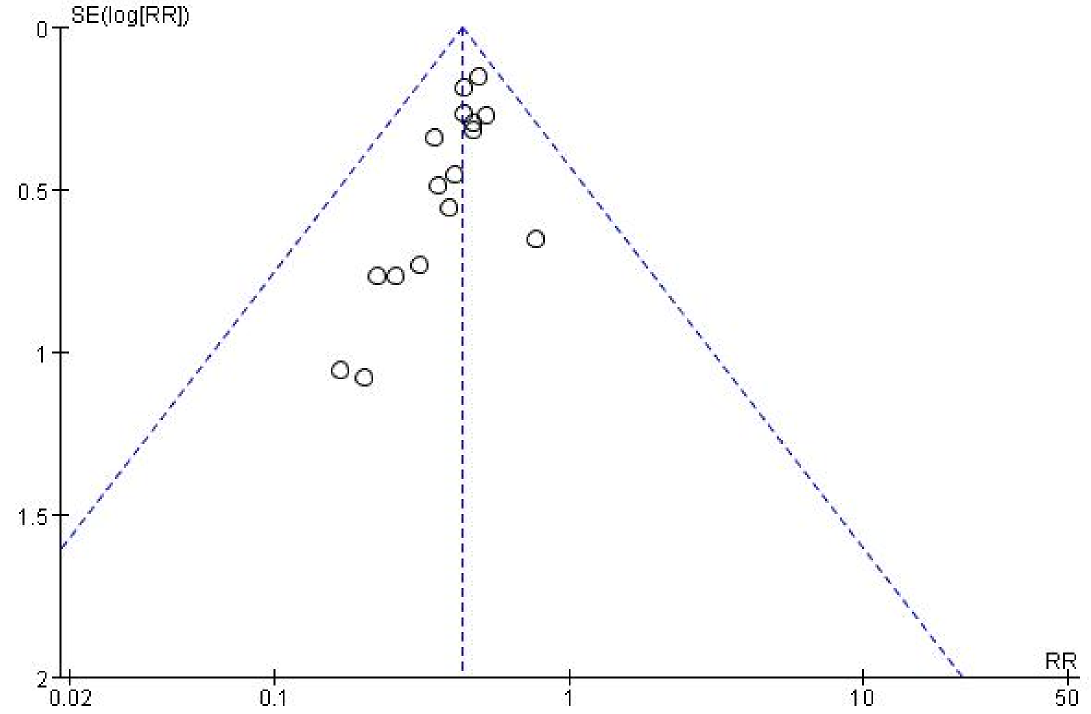

Supplement: Supplementary file 1 [file Data_Sheet_1.pdf]
