# Supplementary material for: Efficacy and safety of vonoprazan-amoxicillin dual therapy versus bismuth-containing quadruple therapy for patients with Helicobacter pylori infection: a meta-analysis
Source: Front Microbiol. 2025 Mar 19;16:1561749. doi: 10.3389/fmicb.2025.1561749 (PMC11962034; doi:10.3389/fmicb.2025.1561749)
Supplement: Supplementary file 2 [file Data_Sheet_2.pdf]

PubMed		
No.	Query	Results
1	Helicobacter pylori[Title/Abstract] OR Helicobacter nemestrinae[Title/Abstract] OR Campylobacter pylori[Title/Abstract] OR Campylobacter pylori[Title/Abstract] OR Campylobacter pyloridis[Title/Abstract]	47923 results
2	Therapeutic[Title/Abstract] OR Therapy[Title/Abstract] OR Therapies[Title/Abstract] OR Treatment[Title/Abstract] OR Treatments[Title/Abstract]	7745865 results
3	Omeprazole[Title/Abstract] OR Lansoprazole[Title/Abstract] OR Lansoprazol[Title/Abstract] OR Lanzor[Title/Abstract] OR Pantoprazole[Title/Abstract] OR Protonix[Title/Abstract] OR Esomeprazole[Title/Abstract] OR Rabeprazole[Title/Abstract] OR Proton-pump inhibitor[Title/Abstract] OR PPI[Title/Abstract]	47436 results
4	Amoxicillin[Title/Abstract] OR Amoxycillin[Title/Abstract] OR Amoxicilline[Title/Abstract] OR Tetracycline[Title/Abstract] OR Tetrabid[Title/Abstract] OR Topicycline[Title/Abstract] OR Furazolidone[Title/Abstract] OR Furazolidine[Title/Abstract] OR Furoxone[Title/Abstract] OR Furazol[Title/Abstract] OR Metronidazole[Title/Abstract] OR Metric[Title/Abstract] OR MetroGel[Title/Abstract] OR Metrogyl[Title/Abstract] OR Clarithromycin[Title/Abstract] OR Levofloxacin[Title/Abstract] OR Quixin[Title/Abstract] OR Levaquin[Title/Abstract]	134910 results
5	Bismuth containing quadruple therapy[Title/Abstract] OR bismuth tripotassium dicitrate[Title/Abstract] OR tripotassium-dicitrato bismuthate[Title/Abstract] OR colloidal bismuth subcitrate[Title/Abstract] OR bismuth subcitrate[Title/Abstract] OR De-Nol[Title/Abstract] OR De-Noltab[Title/Abstract] OR DeNol[Title/Abstract] OR Ventrisol-polfa[Title/Abstract] OR bismuth citrate[Title/Abstract] OR Bi-citrate[Title/Abstract] OR ranitidine bismuth citrate[Title/Abstract] OR Pylorid[Title/Abstract] OR Tritec[Title/Abstract] OR Raniberl[Title/Abstract]	1296 results
6	potassium competitive acid blocker[Title/Abstract] OR Vonoprazan[Title/Abstract] OR Revaprazan hydrochloride[Title/Abstract] OR Revaprazan[Title/Abstract] OR Tegoprazan[Title/Abstract] OR soraprazan[Title/Abstract] OR remofuscin[Title/Abstract] OR Linaprazan[Title/Abstract]	759results
7	1 AND 2 AND 3 AND 4 AND 5 AND 6	11results 2024/6/2

Web of science		
No.	Query	Results
1	((((TS=(Helicobacter pylori)) OR TS=(Helicobacter nemestrinae)) OR TS=(Campylobacter pylori)) OR TS=(Campylobacter pylori)) OR TS=(Campylobacter pyloridis) and Preprint Citation Index (Exclude – Database)	125778 results
2	((((TS=(Therapeutic)) OR TS=(Therapy)) OR TS=(Therapies)) OR TS=(Treatment)) OR TS=(Treatments) and Preprint Citation Index (Exclude – Database)	26130810results
3	(((((((TS=(potassium competitive acid blocker)) OR TS=(Vonoprazan)) OR TS=(Revaprazan hydrochloride)) OR TS=(Revaprazan)) OR TS=(Tegoprazan)) OR TS=(soraprazan)) OR TS=(remofuscin)) OR TS=(Linaprazan) and Preprint Citation Index (Exclude – Database)	1938 results
4	(((((((((TS=(Omeprazole)) OR TS=(Lansoprazole)) OR TS=(Lansoprazol)) OR TS=(Lanzor)) OR TS=(Pantoprazole)) OR TS=(Protonix)) OR TS=(Esomeprazole)) OR TS=(Rabeprazole)) OR TS=(Proton-pump inhibitor)) OR TS=(PPI) and Preprint Citation Index (Exclude – Database)	118260 results
5	((((((((((((((((TS=(Amoxicillin)) OR TS=(Amoxycillin)) OR TS=(Amoxicilline)) OR TS=(Tetracycline)) OR TS=(Tetrabid)) OR TS=(Topicycline)) OR TS=(Furazolidone)) OR TS=(Furazolidine)) OR TS=(Furoxone)) OR TS=(Furazol)) OR TS=(Metronidazole)) OR TS=(Metric)) OR TS=(MetroGel)) OR TS=(Clarithromycin)) OR TS=(Metrogyl)) OR TS=(Levofloxacin)) OR TS=(Quixin)) OR TS=(Levaquin) and Preprint Citation Index (Exclude – Database)	934634 results
6	((((((((((((((TS=(Bismuth containing quadruple therapy)) OR TS=(bismuth tripotassium dicitrate)) OR TS=(tripotassium-dicitrato bismuthate)) OR TS=(colloidal bismuth subcitrate)) OR TS=(bismuth subcitrate)) OR TS=(De-Nol)) OR TS=(De-Noltab)) OR TS=(DeNol)) OR TS=(Ventrisol-polfa)) OR TS=(bismuth citrate)) OR TS=(Bi-citrate)) OR TS=(ranitidine bismuth citrate)) OR TS=(Pylorid)) OR TS=(Tritec)) OR TS=(Raniberl) and Preprint Citation Index (Exclude – Database)	4666 results
7	1 AND 2 AND 3 AND 4 AND 5 AND 6	52 results<br>2024/6/2

Embase		
No.	Query	Results
#1	'helicobacter pylori':ab,ti OR 'helicobacter nemestrinae':ab,ti OR 'campylobacter pylori':ab,ti OR 'campylobacter pyloridis':ab,ti	63880 results
#2	'therapeutic':ab,ti OR 'therapy':ab,ti OR 'therapies':ab,ti OR 'treatment':ab,ti OR 'treatments':ab,ti	10687508 results
#3	'potassium competitive acid blocker':ab,ti OR 'vonoprazan':ab,ti OR 'revaprazan hydrochloride':ab,ti OR 'revaprazan':ab,ti OR 'tegoprazan':ab,ti OR 'soraprazan':ab,ti OR 'remofuscin':ab,ti OR 'linaprazan':ab,ti	1191 results
#4	'omeprazole':ab,ti OR 'lansoprazole':ab,ti OR 'lansoprazol':ab,ti OR 'lanzor':ab,ti OR 'pantoprazole':ab,ti OR 'protonix':ab,ti OR 'esomeprazole':ab,ti OR 'rabeprazole':ab,ti OR 'proton-pump inhibitor':ab,ti OR 'ppi':ab,ti	70251 results
#5	'amoxicillin':ab,ti OR 'amoxycillin':ab,ti OR 'amoxicilline':ab,ti OR 'tetracycline':ab,ti OR 'tetrabid':ab,ti OR 'topicycline':ab,ti OR 'furazolidone':ab,ti OR 'furazolidine':ab,ti OR 'furoxone':ab,ti OR 'furazol':ab,ti OR 'metronidazole':ab,ti OR 'metric':ab,ti OR 'metrogel':ab,ti OR 'metrogyl':ab,ti OR 'clarithromycin':ab,ti OR 'levofloxacin':ab,ti OR 'quixin':ab,ti OR 'levaquin':ab,ti	177178 results
#6	'bismuth containing quadruple therapy':ab,ti OR 'bismuth tripotassium dicitrate':ab,ti OR 'tripotassium-dicitrato bismuthate':ab,ti OR 'colloidal bismuth subcitrate':ab,ti OR 'bismuth subcitrate':ab,ti OR 'de-nol':ab,ti OR 'de-noltab':ab,ti OR 'denol':ab,ti OR 'ventrisol-polfa':ab,ti OR 'bismuth citrate':ab,ti OR 'bi-citrate':ab,ti OR 'ranitidine bismuth citrate':ab,ti OR 'pylorid':ab,ti OR 'tritec':ab,ti OR 'raniberl':ab,ti	1763 results
#7	#1 AND #2 AND #3 AND #4 AND #5 AND #6	16 results<br>2024/6/2

cochrane library		
No.	Query	Results
#1	(((((Helicobacter pylori) OR (Helicobacter nemestrinae)) OR (Campylobacter pylori)) OR (Campylobacter pylori)) OR (Campylobacter pyloridis)):ti,ab,kw	6257 results
#2	((((((Therapeutic)) OR (Therapy)) OR (Therapies)) OR (Treatment)) OR (Treatments)):ti,ab,kw	1308216 results
#3	(potassium competitive acid blocker OR Vonoprazan OR Revaprazan hydrochloride OR Revaprazan OR Tegoprazan OR soraprazan OR remofuscin OR Linaprazan):ti,ab,kw	630 results
#4	(Omeprazole OR Lansoprazole OR Lansoprazol OR Lanzor OR Pantoprazole OR Protonix OR Esomeprazole OR Rabeprazole OR Proton-pump inhibitor OR PPI):ti,ab,kw	11603 results
#5	(Amoxicillin OR Amoxycillin OR Amoxicilline OR Tetracycline OR Tetrabid OR Topicycline OR Furazolidone OR Furazolidine OR Furoxone OR Furazol OR Metronidazole OR Metric OR MetroGel OR Metrogyl OR Clarithromycin OR Levofloxacin OR Quixin OR Levaquin):ti,ab,kw	16845 results
#6	(Bismuth containing quadruple therapy OR bismuth tripotassium dicitrate OR tripotassium-dicitrato bismuthate OR colloidal bismuth subcitrate OR bismuth subcitrate OR De-Nol OR De-Noltab OR DeNol OR Ventrisol-polfa OR bismuth citrate OR Bi-citrate OR ranitidine bismuth citrate OR Pylorid OR Tritec OR Raniberl):ti,ab,kw	1150 results
#7	#1 AND #2 AND #3 AND #4 AND #5 AND #6	43 results<br>2024/6/2
